# Restricted collision coupling of the adenosine A_2A_ receptor is due to its agonist-induced confinement in the membrane

**DOI:** 10.1186/2050-6511-13-S1-A81

**Published:** 2012-09-17

**Authors:** Patrick Thurner, Simon Keuerleber, Ingrid Gsandtner, Christoph Gruber, Michael Freissmuth, Jürgen Zezula

**Affiliations:** 1 Institute of Pharmacology, Center for Physiology and Pharmacology, Medical University of Vienna, 1090 Vienna, Austria

## Background

The A_2A_ adenosine receptor is of interest because of several reasons. (i) It is a frequently blocked pharmacological target, because it is the site of action of caffeine. (ii) It has a long C-terminus that provides a docking site for several proteins, which direct the fate of the receptor from its synthesis to its lysosomal degradation. (iii) The A_2A_ receptor can only promote activation of a limited number of available G_s_ molecules. This coupling mode was termed restricted collision coupling. (iv) Most G protein-coupled receptors carry one or several cysteine residues in their C-terminus which is subject to palmitoylation to anchor and stabilize the amphipathic helix 8; the A_2A_ receptor lacks this palmitoylation site. We explored the hypothesis that there is a causal link between the absence of a palmitoyl moiety and restricted collision coupling.

## Methods

We constructed a mutant A_2A_ receptor, R309C, which underwent palmitoylation as verified by both mass spectrometry and metabolic labeling. Radioligand binding, cAMP accumulation and Western blotting were performed to determine its signaling properties. Using single particle tracking of quantum dot-labeled receptors we compared diffusivity and diffusion mode of wild-type and mutant A_2A_ receptors.

## Results

In contrast to the wild-type receptor, the concentration-response curve for agonist-induced cAMP accumulation was shifted to the left with increasing expression levels of A_2A_ receptor R309C. Single particle tracking demonstrated that agonist activation resulted in a decline in mean square displacement of both receptors, but the drop was substantially more pronounced for the wild-type receptor. In addition, in the agonist-bound state, the wild-type receptor was frequently subject to confinement events; these were rarely seen with the palmitoylated A_2A_ receptor R309C.

## Conclusions

Taken together, the observations link restricted collision coupling to diffusion limits imposed by the absence of a palmitoyl moiety in the C-terminus of the A_2A_ receptor.

